# Demonstrative Midwifery

**Published:** 1850-08

**Authors:** 


### EDITORIAL DEPARTMENT.

*Demonstrative Midwifery. Report of the Trial: The People versus Dr. Horatio N. Loomis for Libel. Tried at the Erie County Oyer and Terminer, June* 24, 1850. *Justice Mullett, Presiding. John Treanor, and Leander J. Roberts, Associate Justices. Reported by Jesse Walker, Esq., assisted by Mr. Frederick T. Parsons, Stenographer. Buffalo: Steam Press of Jewett, Thomas & Co.* 1850.

It was our intention, as intimated in our last No., to have copied from the published report of the trial of Dr. Loomis, that portion of the testimony developing the facts connected with the demonstration of labor at the Buffalo Medical College during the last session, and also the testimony of the medical witnesses summoned on the part of the people, and for the defence. In order, however, that this subject may not encroach too much upon space which many might desire should be devoted to other matters, we have arranged for having a copy of the report sent to each of the subscribers to the Buffalo Medical Journal. This will render it unnecessary to carry out the plan we had intended.

We also announced a design of reviewing the facts, and the testimony, evolved by the trial referred to, discussing the validity of the objections made to demonstrative teaching in midwifery, and, at the same time, examining its advantages. But, we confess, we are somewhat at a loss to determine in how far it is advisable to carry the latter resolution into effect. Our indecision springs from the following considerations: The facts and the testimony appear to us to speak sufficiently for themselves, as contained in the published report of the trial. Then, as regards the merits of the subject incidentally connected with the trial, to wit, *demonstrative midwifery*, it really seems to us that they hardly call for labored argumentation, but that they at once suggest and commend themselves. Moreover, we have every reason to conclude that with nine-tenths of the medical profession the claims of demonstrative teaching in midwifery require neither exposition nor advocacy. If we are in error on this point we are ready to be corrected, and to acknowledge that we have erred. But the subject has now been before the medical public several months, and, as yet, no one, that we are aware of, has come forward to sustain the assertion that demonstrative teaching in midwifery is *unprofessional,** *wholly unnecessary, immoral, and indecent.* Of some fifteen medical Journals of the United States, at least ten have already contained decided expressions of approbation of this method of teaching; and this has not called out from the ranks of the profession, as yet, even a solitary knight to do battle in behalf of those who think it should not be practiced "*in any civilized community!*"† None of those who thought it their duty to administer a "*severe rebuke,*"‡ appear to think the attitude they have assumed worth contending for. We have invited communications on the subject from those who oppose, as well as those who approve of demonstrative midwifery, but, to our regret, the discussion is left to ourselves alone. We say "to our regret"—we have no fondness for controversy, but, under present circumstances, we are not reluctant to discuss this subject in any of its aspects or bearings; and we persuade ourselves that we are able to treat it with candor, notwithstanding, in connection therewith, as a member of the Faculty of the Medical College of Buffalo, we stand charged by seventeen of our professional neighbors with having participated in an immoral and indecent act. In view of this serious accusation, we claim that what we have written on this subject has been temperate, to say the least; nor have we any disposition to recriminate by inquiring into motives, so long as there seems no necessity for resorting to a method of self-protection so uncongenial.

In view of the circumstances just stated, we repeat, we are at a loss to know what degree of consideration we should give to the subject at this time, wishing to avoid, on the one hand, needlessly taxing the patience of our readers, and, on the other hand, resolved to be completely relieved of imputations which we cannot but regard as alike uncalled for, unjust, and ungenerous. In this state of uncertainty we purpose to pursue a middle course, noticing the facts and testimony briefly, and commenting on a few only of the points which a full discussion of the subject would involve.

---

* See letter of seventeen physicians of Buffalo, in the March No. of this Journal, 1850.

† See ibid.

‡ Ibid.

First, as to the *facts.* If the reader will peruse carefully the testimony of those of the witnesses who were present at the demonstration, he will find that the details, in so far as we have professed to state of them in our previous notices, are fully substantiated. On one point we have hitherto withheld entering into a specific statement. This is the degree of exposure. We have avoided speaking of that minutely, in the first place, because, in so far as our own opinion is concerned, we hold it to be a point of very little consequence. We wish it to be distinctly understood, that we advocate Demonstrative Midwifery on the ground of its intrinsic propriety and utility, and that any exposure which is useful, is, in our view, perfectly proper. But, since so much importance appears to be attached to this point by those who claim the prerogative of administering a rebuke, we preferred to delay for the evidence given under oath. And what do the witnesses testify on this point? *They all of them swear that they did not see the genitals of the patient, which were protected from view by the cautious management of the Professor of obstetrics.* We refer to the report of the trial for proof of the correctness of this assertion. The exposure was limited to the head of the child, and a small portion, perhaps, of the *nates*, or hips of the woman. We do not adduce this fact as one for which the Professor in charge of the demonstration is entitled to credit. We think with our esteemed colleague Prof. Lee, (see his testimony,) that enough was not exhibited; the demonstration was, in our opinion, defective in this respect. And we take occasion to say, that when the matter was submitted to the members of the Faculty of the College, it was expected that the exposure would be more complete. We shall not complain of the over prudence of the Professor of obstetrics, but we think the demonstration is susceptible of an improvement by which the students would have a fuller opportunity to witness the changes which the perineum undergoes in the process of the delivery.

Another highly interesting fact appears in the trial, to which we have not before alluded. It seems that at the stethoscopic examinations, several days prior to the labor, the Professor of obstetrics had predicted, from the situation of the fœtal sounds, an unusual position of the fœtus, viz., an anterior direction of the face. The correctness of this opinion was verified at the labor; and the fact that the prediction had been publicly pronounced, furnished an inducement to exhibit to the class the passage of the head through the vulva, that they might the better realize the accuracy of the deduction from the auscultatory phenomena. Assuming, for the moment, that, in ordinary cases, ocular demonstrations in midwifery are neither useful nor proper for purposes of teaching, a generous mind, it would seem, could overlook an exposure so guarded as in this instance, and with the full consent of the patient, from sympathy with the desire of a teacher to test the precision of diagnosis, even were it but to indulge a little pride of professional acumen; but, still more, when, at the same time, it would serve to impress most vividly upon the mind of the student a sense of the reality and practical utility of the art of obstetrical auscultation. Let us not be understood to offer this as an apology for the demonstration in this instance. In no point of view do we concede, for an instant, that a demonstration of labor, properly conducted, for purposes of instruction, requires a word of apology.

We pass now to the medical testimony relative to the utility and propriety of Demonstrative Midwifery. On the part of the Defence, fifteen medical witnesses were examined, who testified that, in their opinions, demonstrative teaching of midwifery is *unnecessary* and *improper*. The reader, by comparing the names of these witnesses with those attached to the letter contained in the March No. of this Journal, will perceive that all but three of the former are enrolled among the latter. Now, as a commentary on the merits of the subject, we ask the reader to peruse carefully the direct and cross examinations of these witnesses. Each and all are persuaded that nothing is gained for the pupil by an ocular exhibition of the visible stage of labor. Yet there is no one of them that hesitates to testify to the great utility of drawings, pictures, and models, designed to illustrate this stage. Most valuable information can be derived by the *representation* of living parts, but they can perceive no advantages in seeing the *living parts themselves*. Not one of them recognizes the least indelicacy in studying the processes of labor in so far as they can be delineated on paper, or canvass, or imitated by wax, *papier mache*, and buckskin, and the more natural and life-like these resemblances are, of course, the better; but to exhibit the same parts and processes as nature herself displays them, is useless and improper! It is well to study by the reflected rays of the *moon*, but quite superfluous to avail ourselves of the light of the *sun!* The *shadow* cannot be dispensed with, but the *substance* is of no avail! We should be fearful of being suspected of exaggeration, if the reader were not supposed to have the report of the trial before him. He will find on reading the testimony, that the metaphors we have just used are scarcely hyperbolical. Several swear that plates and drawings are quite as useful for instruction as the real objects which they represent; one, at least, thinks they are even *better* than nature, and one witness testifies that comparative anatomy may furnish as useful demonstrations as the living subject! This is certainly an idea which is entitled to the merit of originality. Imagine the Faculty of the Medical College to have acted upon such a suggestion, and procured for the class an opportunity to witness the parturition, for example, of a bovine quadruped! But, seriously, does the projector of this new method of teaching obstetrics really think any useful ends are to be gained by it? then, plainly, he concedes all that is claimed for demonstrative midwifery, unless he contends that an inferior animal affords a *better* illusiration of labor in the human subject, than the human subject herself!

The reader will observe that much is said by the witnesses for the defence of the importance of cultivating the sense of touch. No one denies the importance of this, but will it be deemed otherwise than absurd that because the touch is to be informed, the sight is useless? Do we not better educate one sense by associating with it knowledge derived by other senses? It is true, when a sense is lost, some of the other senses are cultivated with greater assiduty, so as to compensate in some degree for the deficiency; but will it be gravely argued from this fact, that the different senses, more particularly the sight and touch, are not instrumental in educating each the others? According to this notion, blindness would be a good qualification for an accoucheur! But their own testimony disproves their notions, for all admit the utility of plates, drawings, and manikins, and is not all the information therefrom derived addressed exclusively to the eye!

And why this distinction among the senses as regards *impropriety* in their use? If there be indelicacy in *seeing,* is there not the same, or even more, in *touching!* If the possibility of improper emotions is to be entertained, (and God forbid that it should be for a moment,) is the sense of *feeling* less capable of administering thereto than that of *sight!* Yet, with a single exception, all these witnesses testify, not only to the usefulness, but to the *propriety* of permitting medical students to make vaginal examinations for purposes of instruction.

But demonstrative teaching of midwifery *shocks the moral sense of the community.* We do not admit this except for argument's sake. Were it so, we ask if the moral sense of the community is to be taken as the guide and umpire in questions relating to proposed improvements in medical Instruction? Do we defer to the moral sense of the community in other measures: if so, why are not dissections legalized by the popular will? We say we do not admit that demonstrative teaching in midwifery does shock the moral sense of the community. We believe, at this moment, in this city, where the subject has been agitated, that the predominance of public sentiment is in favor of it; and, yet, a community would hardly be expected to be in favor of a novel mode of medical teaching which a number of medical men residing in that community had united in publicly denouncing as unprofessional, wholly unnecessary, offensive to morality and common decency.

On behalf of the prosecution, seventeen witnesses were examined, who testified in favor of the utility and propriety of demonstrative midwifery. We do not propose to analyze the testimony of these witnesses; but in commending it to the reader's attention we will simply allude to two or three points. Of the witnesses examined on either side, in the trial of this case, several had been practically acquainted with demonstrations in midwifery, and several had not seen a single demonstration. Now of all those embraced in the first category, not one testified to the inutility of this method of teaching; and of those who testified to its being unnecessary and improper, not one had any practical acquaintance with it. This is worthy of mention as a significant fact.

Again, of the members of the graduating class, now practising physicians, who witnessed the demonstration, all who were examined on the part of the people, and by the defence, testified that it had been of service to them—that they had gained information, and were better prepared by it to enter on the practical duties of the accoucheur. Now they could hardly be mistaken on this point. They testified to that which they did *know*. And in matters of this kind, so far as the authority of medical opinion is concerned, their testimony is worth infinitely more than that of older members of the profession, who may be supposed to have forgotten, in part, the difficulties and embarrassments incident to their first practical efforts in midwifery. This, as it seems to us, is an obvious truth, and in no wise disparages the respect due to age and experience in matters of a different nature.

Again, we claim that medical teachers should be expected to understand the necessities and interests of medical pupils somewhat better than those who have had little or no practical experience in medical instruction. In saying this, we assume no more than that a man who devotes a good share of his time and attention to any pursuit, ought to judge best of matters relating to that pursuit, and his opinions are entitled to additional weight from that circumstance. In view of this consideration, the reader of the trial will doubtless attach especial importance to the testimony of Professor

Gilman, of the city of New York, teacher of obstetrics in the oldest, and, we may add, without invidiousness, the *first* medical institution of this State; and to that of Prof. Coventry, also an eminent, and, we may add, a veteran teacher in the same department of medical instruction. We mention these names because the subject under remark relates to a branch of medical science which it has been the business of their lives to study and expound.

On the part of the defence, it will be observed no person connected with a medical school, or who professed to be engaged in medical teaching, appeared to testify *against* the utility and propriety of demonstrative midwifery. The reader will deem it hardly probable that such witnesses would not have been summoned, were they to be found, especially when he observes the proposition by the defendant's counsel, to introduce several *matrons* or *midwives*, who had been subpœned, to testify as to the necessity of exposure in teaching obstetrics! (See page 17 of the report.) This was certainly a singular proposition, and highly complimentary to the professional opinions that had preceded. The court, however, refused to recognize them as *professional persons*, and, hence, we have not the advantage of their testimony.

The advantages of demonstrative teaching in midwifery are not developed in the testimony so fully as to do adequate justice to them, the matter at issue in the trial, i. e. the libel, having little to do with an elaborate exposition of all the merits of the subject. What was not elicited by the course of examination pursued, however, the reflections of the reader will doubtless supply. It is only necessary to go back and review one's primary experience in obstetrics to appreciate the need of clinical and demonstrative instruction. The retrospections of many of our readers will speak to their minds more forcibly on this point than any language we could use. The first case of labor, at the commencement of a professional career, is not only an occasion full of embarrassment and apprehension, but often an event of momentous importance to the young practitioner, if not to others. His theoretical knowledge is to be put to a practical test; the shrewd and suspicious eyes of patient, nurse, and neighbors, are watching all his movements; he may be asked if he has ever attended on a similar occasion, and shall he equivocate or falsify? More than this, the very first case may present unusual difficulties, which call, if not for experience, at least calmness and *self-possession.* Will any one deliberately deny that under any or all these circumstances, a person who has witnessed the details of one or more labors conducted by his instructor, will have more confidence, and be more competent to discharge his duties to the patient, to satisfy the expectations of friends and attendants, and advance his professional success? But it may be said by some of those who have entered their protest against demonstrative midwifery, we do not object to any kind of clinical teaching which is conducted without exposure. They may perhaps approve of all that was done at the medical college, except the display of the head of the child, a portion of the nates and hips of the patient for the space of from two to five minutes, the room being illuminated with one or two tallow candles! This is the objectionable feature of the demonstration, which their outraged feelings compelled them to denounce, forgetting, at the same time, to signify their approbation of all the rest, and the credit due to the Professor of Obstetrics, and the Faculty, for having supplied unusual facilities for clinical instruction, irrespective of this alledged violation of propriety!

Had we time and space, we should dwell somewhat upon the advantages of this particular part of the demonstration; although we cannot but think it would be supererogatory, if the reader will consider that it is precisely at the stage of labor when the exhibition was made, i. e., when the head emerges from the vulva, that, in ordinary cases, the services of the accoucher are required, and these services have exclusive reference to what was exhibited in the passage of the child through the *os externum.* The perineal tumour, the dilatation which the perineum undergoes, the mode and direction in which it is to be supported, the curvilinear movement of the fœtal head, its rotation while the body is passing through the pelvis, etc. — all these points are not only minutely and carefully described in books and lectures, but it is always expected will also be taught by *illustrations.* And will it seriously be said that the *living* subject does not present better illustrations than *drawings, pictures,* and a *buckskin manikin!* Will the latter produce in the mind of the pupil as correct an idea, and as vivid, forcible impression as the appearances and operations of nature herself!

There is another view of this subject, which, if this article were not already so extended, we should consider. In all our remarks hitherto, we have spoken of demonstrations in midwifery in connection with their utility in preparing for the practical duties of the medical practitioner. But we are prepared to take broader ground than this, and to say, that, in our opinion, they are not offensive either to morality or decency, irrespective of practical instruction. We do not hesitate to avow our belief not only that such demonstrations might be witnessed by the physiologist, the philosopher, the moralist, and the christian without detriment to the moral or religious sense, albeit the observer might never intend to become a medical practitioner, but that even the thoughtless and licentious man would find in them nothing to foster base or unworthy sentiments. We challenge any respectable medical man to come forward and declare that the lying-in chamber is a place in which to harbor thoughts of lewdness or frivolity. Every physician and every reflecting man, medical or not, must know that it is calculated to have a far different moral influence. We pity him, if there be such an one, who can think otherwise, and still more, one who does not blush to give utterance to such a thought — a thought which is insulting to all that is compassionate and manly in human character. Nay, more, it is insulting to Divine Providence, for there is truth as well as wit, in the following pithy remark by our friend, Prof. Ackley, of Cleveland: "It is impugning the wisdom of the Creator to say that He has ordained children to be born into the world in a way not fit to be seen."

If to any of our readers it may seem that we have dwelt too much on Demonstrative Midwifery in this, and former numbers of our Journal, we beg, as our apology, to refer them to the letter on this subject contained in the number for March last, denouncing a measure, for which, in common with our colleagues of the Medical College, we are responsible, as *unprofessional, immoral,* and *indecent.* We would willingly think that the letter referred to was penned under the influence of erroneous representations as to the facts in the case, and that it was signed without duly weighing the force of the language used, and under the impression that its object was merely to disapprove of the innovation, and to dissent from the editorial opinion advanced in the preceding number of this Journal. Indeed, we may say that some of the signers of that letter have signified thus much. But the public *rebuke* has not been formally withdrawn, modified or explained. We cannot but think that a medical association would have been a more appropriate place to have preferred such an accusation, but since, in lieu of this, the *medical public* has been appealed to, we shall look to that tribunal for an acquittal. Farther than this, we have no feelings to gratify, or personal ends to attain by prolonging a discussion of the subject.

In closing this article, we would commend to the attention of the reader the admirable charge by Justice Mullett, and the abstract of the masterly argument by Hon. H. K. Smith, which are contained in the report of the trial. It is to be regretted that notes of the ingenious and successful plea of Henry W. Rogers, Esq. were not taken, so that the publication might have been still more complete. The opening speech for the defence by

Mr. Putnam, a spirited and forcible effort, is embraced in the report Appended to the report are articles from the Medical Journals that have contained expressions of opinion relative to demonstrative midwifery, and to the action taken by members of the medical profession of this city on the subject. We had promised to copy these articles, but since they are contoined in the publication of the trial, and will thus be in the hands of all our readers, their insertion in our Journal will be dispensed with. It were gratuitous to commend them to our readers.

